# Supplementary material for: Machine learning identification of Pseudomonas aeruginosa strains from colony image data
Source: PLoS Comput Biol. 2023 Dec 13;19(12):e1011699. doi: 10.1371/journal.pcbi.1011699 (PMC10752536; doi:10.1371/journal.pcbi.1011699)
Supplement: S1 Fig — Morphological (green) and complexity (orange) metrics. (DOCX) [file pcbi.1011699.s001.docx]

Supplemental Figure S1. Correlation matrix for all morphological and complexity metrics. Morphological (green) and complexity (orange) metrics.
