# Supplementary material for: Machine learning identification of Pseudomonas aeruginosa strains from colony image data
Source: PLoS Comput Biol. 2023 Dec 13;19(12):e1011699. doi: 10.1371/journal.pcbi.1011699 (PMC10752536; doi:10.1371/journal.pcbi.1011699)
Supplement: S1 Table — The performance of four trained transfer learning models (ResNet-50, VGG-19, MobileNetV2 and Xception, see methods) were evaluated on both validation and test datasets. Each computational experiment was replicated five-fold (five separate training runs for each model), allowing statistical comparison of approaches. Table A. Summary statistics. Across replicates we report average (+/- standard deviation) accuracy (the number of correct predictions divided by the total number of predictions x100) and loss (a summation of the errors made for each sample). Table B. ANOVA table. Tables C-F. Post-hoc pairwise tests (Tukey HSD with alpha = 0.05). (DOCX) [file pcbi.1011699.s002.docx]

**S1 Table. Statistical comparisons of transfer learning methods (Fig 5).** The performance of four trained transfer learning models (ResNet-50, VGG-19, MobileNetV2 and Xception, see methods) were evaluated on both validation and test datasets. Each computational experiment was replicated five-fold (five separate training runs for each model), allowing statistical comparison of approaches.

**Table A. Summary statistics.** Across replicates we report average (+/- standard deviation) accuracy (the **number of correct predictions divided by the total number of predictions x100) and loss (**a summation of the errors made for each sample).

| **Transfer learning Method** | **Validation accuracy (%)** | **Validation loss** | **Test accuracy (%)** | **Test loss** |
| --- | --- | --- | --- | --- |
| ResNet 50 | 92.93 ± 4.831 | 0.251 ± 0.174 | 90.73 ± 5.073 | 0.278 ± 0.183 |
| VGG-19 | 86.96 ± 4.033 | 0.448 ± 0.130 | 83.92 ± 4.232 | 0.510 ± 0.137 |
| MobileNetV2 | 54.40 ± 6.252 | 1.853 ± 0.303 | 53.39 ± 6.563 | 2.043 ± 0.318 |
| Xception | 90.25 ± 4.561 | 0.300 ± 0.201 | 88.36 ± 4.788 | 0.330 ± 0.210 |

**Table B. ANOVA table.**

| **ANOVA** | **sum_sq** | **df** | **F** | **P-value** |
| --- | --- | --- | --- | --- |
| Validation Accuracy | 4700.077255 | 3.0 | 119.531973 | 3.627413e-11 |
| Test Accuracy | 4571.131895 | 3.0 | 124.245285 | 2.698834e-11 |
| Validation Loss | 8.867851 | 3.0 | 269.70656 | 6.616010e-14 |
| Test Loss | 10.635474 | 3.0 | 942.817267 | 3.337569e-18 |

**Tables C-F. Post-hoc pairwise tests** (Tukey HSD with alpha = 0.05).

| **Table C. Post Hoc Analysis for Validation Loss** | | | | | | |
| --- | --- | --- | --- | --- | --- | --- |
| **group1** | **group2** | **meandiff** | **p-adj** | **lower** | **upper** | **reject** |
| MobileNetV2 | ResNet-50 (Baseline) | -1.6076 | 0.0 | -1.797 | -1.4182 | True |
| MobileNetV2 | VGG-19 | -1.4158 | 0.0 | -1.6052 | -1.2264 | True |
| MobileNetV2 | Xception | -1.5636 | 0.0 | -1.753 | -1.3742 | True |
| ResNet-50 (Baseline) | VGG-19 | 0.1918 | 0.0467 | 0.0024 | 0.3812 | True |
| ResNet-50 (Baseline) | Xception | 0.044 | 0.9088 | -0.1454 | 0.2334 | False |
| VGG-19 | Xception | -0.1478 | 0.1567 | -0.3372 | 0.0416 | False |
| **Table D. Post Hoc Analysis for Test Loss** | | | | | | |
| **group1** | **group2** | **meandiff** | **p-adj** | **lower** | **upper** | **reject** |
| MobileNetV2 | ResNet-50 (Baseline) | -1.7704 | 0.0 | -1.8814 | -1.6594 | True |
| MobileNetV2 | VGG-19 | -1.5342 | 0.0 | -1.6452 | -1.4232 | True |
| MobileNetV2 | Xception | -1.7116 | 0.0 | -1.8226 | -1.6006 | True |
| ResNet-50 (Baseline) | VGG-19 | 0.2362 | 0.0001 | 0.1252 | 0.3472 | True |
| ResNet-50 (Baseline) | Xception | 0.0588 | 0.4513 | -0.0522 | 0.1698 | False |
| VGG-19 | Xception | -0.1774 | 0.0016 | -0.2884 | -0.0664 | True |
| **Table E. Post Hoc Analysis for Validation Accuracy** | | | | | | |
| **group1** | **group2** | **meandiff** | **p-adj** | **lower** | **upper** | **reject** |
| MobileNetV2 | ResNet-50 (Baseline) | 37.688 | 0 | 31.1371 | 44.2389 | True |
| MobileNetV2 | VGG-19 | 32.128 | 0 | 25.5771 | 38.6789 | True |
| MobileNetV2 | Xception | 35.502 | 0 | 28.9511 | 42.0529 | True |
| ResNet-50 (Baseline) | VGG-19 | -5.56 | 0.1115 | -12.1109 | 0.9909 | False |
| ResNet-50 (Baseline) | Xception | -2.186 | 0.7763 | -8.7369 | 4.3649 | False |
| VGG-19 | Xception | 3.374 | 0.4751 | -3.1769 | 9.9249 | False |
| **Table F.** **Post Hoc Analysis for Test Accuracy** | | | | | | |
| **group1** | **group2** | **meandiff** | **p-adj** | **lower** | **upper** | **reject** |
| MobileNetV2 | ResNet-50 (Baseline) | 37.13 | 0 | 30.7933 | 43.4667 | True |
| MobileNetV2 | VGG-19 | 31.206 | 0 | 24.8693 | 37.5427 | True |
| MobileNetV2 | Xception | 35.342 | 0 | 29.0053 | 41.6787 | True |
| ResNet-50 (Baseline) | VGG-19 | -5.924 | 0.0711 | -12.2607 | 0.4127 | False |
| ResNet-50 (Baseline) | Xception | -1.788 | 0.8501 | -8.1247 | 4.5487 | False |
| VGG-19 | Xception | 4.136 | 0.2801 | -2.2007 | 10.4727 | False |
