# Supplementary material for: Machine learning identification of Pseudomonas aeruginosa strains from colony image data
Source: PLoS Comput Biol. 2023 Dec 13;19(12):e1011699. doi: 10.1371/journal.pcbi.1011699 (PMC10752536; doi:10.1371/journal.pcbi.1011699)
Supplement: S2 Table — To assess contributions we took the trained ResNet-50 model (Fig 5 and Table A in S1 Table) as a baseline method, and assessed the impact of removing components of our methods pipeline. Five-fold replicated results are summarized in Table 1. Table A. ANOVA table of data in Table 1. Tables B-E. Post-hoc pairwise tests (Tukey HSD with alpha = 0.05). (DOCX) [file pcbi.1011699.s003.docx]

**S2 Table. Performance contribution of data pre-processing, augmentation and training.** To assess contributions we took the trained ResNet-50 model (Fig 5 and Table A in S1 Table) as a baseline method, and assessed the impact of removing components of our methods pipeline. Five-fold replicated results are summarized in Table 1.

**Table A. ANOVA table of data in Table 1.**

| **ANOVA** | **sum_sq** | **df** | **F** | **P-value** |
| --- | --- | --- | --- | --- |
| Validation Accuracy | 14795.48945 | 9.0 | 278.010813 | 3.655742e-33 |
| Test Accuracy | 14972.050258 | 3.0 | 241.435997 | 5.808255e-32 |
| Validation Loss | 30.322865 | 3.0 | 782.2722 | 4.790182e-42 |
| Test Loss | 40.431932 | 3.0 | 977.413531 | 5.719771e-44 |

**Table B-E. Post-hoc pairwise tests (Tukey HSD with alpha = 0.05).**

| Table B. Post Hoc Analysis for Validation Loss | | | | | | |
| --- | --- | --- | --- | --- | --- | --- |
| **Group 1** | **Group 2** | **Meandiff** | **p-adj** | **Lower** | **Upper** | **Reject** |
| No pre-trained weights | Resnet-50 | -2.0578 | 0 | -2.1968 | -1.9188 | TRUE |
| Remove all augmentation | Resnet-50 | -0.457 | 0 | -0.596 | -0.318 | TRUE |
| Remove brightness augmentation | Resnet-50 | -0.3572 | 0 | -0.4962 | -0.2182 | TRUE |
| Remove horizontal flip augmentation | Resnet-50 | -2.0254 | 0 | -2.1644 | -1.8864 | TRUE |
| Remove image normalization | Resnet-50 | -0.457 | 0 | -0.596 | -0.318 | TRUE |
| Remove rotation augmentation | Resnet-50 | -0.3844 | 0 | -0.5234 | -0.2454 | TRUE |
| Remove shear augmentation | Resnet-50 | -0.4578 | 0 | -0.5968 | -0.3188 | TRUE |
| Remove shift augmentation | Resnet-50 | -0.5098 | 0 | -0.6488 | -0.3708 | TRUE |
| Remove zoom augmentation | Resnet-50 | -2.0628 | 0 | -2.2018 | -1.9238 | TRUE |
| Table C. Post Hoc Analysis for Test Loss | | | | | | |
| **Group 1** | **Group 2** | **Meandiff** | **p-adj** | **Lower** | **Upper** | **Reject** |
| No pre-trained weights | Resnet-50 | -2.5482 | 0 | -2.6917 | -2.4047 | TRUE |
| Remove all augmentation | Resnet-50 | -0.532 | 0 | -0.6755 | -0.3885 | TRUE |
| Remove brightness augmentation | Resnet-50 | -1.3998 | 0 | -1.5433 | -1.2563 | TRUE |
| Remove horizontal flip augmentation | Resnet-50 | -2.082 | 0 | -2.2255 | -1.9385 | TRUE |
| Remove image normalization | Resnet-50 | -0.532 | 0 | -0.6755 | -0.3885 | TRUE |
| Remove rotation augmentation | Resnet-50 | -0.4308 | 0 | -0.5743 | -0.2873 | TRUE |
| Remove shear augmentation | Resnet-50 | -0.5532 | 0 | -0.6967 | -0.4097 | TRUE |
| Remove shift augmentation | Resnet-50 | -0.609 | 0 | -0.7525 | -0.4655 | TRUE |
| Remove zoom augmentation | Resnet-50 | -2.5482 | 0 | -2.6917 | -2.4047 | TRUE |
| Table D. Post Hoc Analysis for Validation Accuracy | | | | | | |
| **Group 1** | **Group 2** | **Meandiff** | **p-adj** | **Lower** | **Upper** | **Reject** |
| No pre-trained weights | Resnet-50 | 59.004 | 0 | 53.8552 | 64.1528 | TRUE |
| Remove all augmentation | Resnet-50 | 52.004 | 0 | 46.8552 | 57.1528 | TRUE |
| Remove brightness augmentation | Resnet-50 | 12.416 | 0 | 7.2672 | 17.5648 | TRUE |
| Remove horizontal flip augmentation | Resnet-50 | 17.94 | 0 | 12.7912 | 23.0888 | TRUE |
| Remove image normalization | Resnet-50 | 16.888 | 0 | 11.7392 | 22.0368 | TRUE |
| Remove rotation augmentation | Resnet-50 | 13.704 | 0 | 8.5552 | 18.8528 | TRUE |
| Remove shear augmentation | Resnet-50 | 19.738 | 0 | 14.5892 | 24.8868 | TRUE |
| Remove shift augmentation | Resnet-50 | 21.358 | 0 | 16.2092 | 26.5068 | TRUE |
| Remove zoom augmentation | Resnet-50 | 18.738 | 0 | 13.5892 | 23.8868 | TRUE |
| Table E. Post Hoc Analysis for Test Accuracy | | | | | | |
| **Group 1** | **Group 2** | **Meandiff** | **p-adj** | **Lower** | **Upper** | **Reject** |
| No pre-trained weights | Resnet-50 | 62.322 | 0 | 56.7641 | 67.8799 | TRUE |
| Remove all augmentation | Resnet-50 | 55.274 | 0 | 49.7161 | 60.8319 | TRUE |
| Remove brightness augmentation | Resnet-50 | 38.774 | 0 | 33.2161 | 44.3319 | TRUE |
| Remove horizontal flip augmentation | Resnet-50 | 22.868 | 0 | 17.3101 | 28.4259 | TRUE |
| Remove image normalization | Resnet-50 | 21.726 | 0 | 16.1681 | 27.2839 | TRUE |
| Remove rotation augmentation | Resnet-50 | 18.506 | 0 | 12.9481 | 24.0639 | TRUE |
| Remove shear augmentation | Resnet-50 | 24.6 | 0 | 19.0421 | 30.1579 | TRUE |
| Remove shift augmentation | Resnet-50 | 26.584 | 0 | 21.0261 | 32.1419 | TRUE |
| Remove zoom augmentation | Resnet-50 | 23.264 | 0 | 17.7061 | 28.8219 | TRUE |
