# Supplementary material for: Machine learning identification of Pseudomonas aeruginosa strains from colony image data
Source: PLoS Comput Biol. 2023 Dec 13;19(12):e1011699. doi: 10.1371/journal.pcbi.1011699 (PMC10752536; doi:10.1371/journal.pcbi.1011699)
Supplement: S3 Table — (DOCX) [file pcbi.1011699.s004.docx]

**S3 Table.** Parameters for all models.

**Model 1: ResNet50-based Classifier**

- **Pre-trained model:** ResNet50
- **Input image dimensions:** 224x224
- **Batch size:** 32
- **Additional model layers:**
  - Flatten layer
  - Fully connected layer with 512 units and ReLU activation
  - Dropout with a rate of 0.5
  - Fully connected layer with 256 units and ReLU activation
  - Dropout with a rate of 0.5
  - Output layer with 69 units and softmax activation
- **Optimizer:** Adam with learning rate of 0.0001
- **Loss function:** Categorical Crossentropy
- **Training epochs:** Not specified for this model

**Model 2: MobileNetV2-based Classifier**

- **Pre-trained model:** MobileNetV2
- **Input image dimensions:** 224x224
- **Batch size:** 32
- **Additional model layers:**
  - Flatten layer
  - Fully connected layer with 512 units and ReLU activation
  - Dropout with a rate of 0.5
  - Fully connected layer with 256 units and ReLU activation
  - Dropout with a rate of 0.5
  - Output layer with 69 units and softmax activation
- **Optimizer:** Adam with learning rate of 0.0001
- **Loss function:** Categorical Crossentropy
- **Training epochs:** 120 epochs

**Model 3: VGG19-based Classifier**

- **Pre-trained model:** VGG19
- **Input image dimensions:** 224x224
- **Batch size:** 32
- **Additional model layers:**
  - Flatten layer
  - Fully connected layer with 512 units and ReLU activation
  - Dropout with a rate of 0.5
  - Fully connected layer with 256 units and ReLU activation
  - Dropout with a rate of 0.5
  - Output layer with 69 units and softmax activation
- **Optimizer:** Adam with learning rate of 0.0001
- **Loss function:** Categorical Crossentropy
- **Training epochs:** 120 epochs

**Model 4: Xception-based Classifier**

- **Pre-trained model:** Xception
- **Input image dimensions:** 299x299
- **Batch size:** 32
- **Additional model layers:**
  - Global Average Pooling layer
  - Batch Normalization layer
  - Fully connected layer with 512 units and ReLU activation
  - Dropout with a rate of 0.3
  - Fully connected layer with 256 units and ReLU activation
  - Dropout with a rate of 0.3
  - Output layer with 69 units and softmax activation
- **Optimizer:** Adam with learning rate of 0.0001
- **Loss function:** Categorical Crossentropy
- **Training epochs:** 120 epochs

**Model 4: Xception-based Classifier**

- **Pre-trained model:** Xception
- **Input image dimensions:** 299x299
- **Batch size:** 32
- **Additional model layers:**
  - Global Average Pooling layer
  - Batch Normalization layer
  - Fully connected layer with 512 units and ReLU activation
  - Dropout with a rate of 0.3
  - Fully connected layer with 256 units and ReLU activation
  - Dropout with a rate of 0.3
  - Output layer with 69 units and softmax activation
- **Optimizer:** Adam with learning rate of 0.0001
- **Loss function:** Categorical Crossentropy
- **Training epochs:** 120 epochs

**Model 5 Configuration:**

- **Model structure:** ResNet50 (without pre-trained ImageNet weights)
- **Input image dimensions:** 224x224
- **Batch size:** 32
- **Additional model layers:**
  - Flatten layer
  - Fully connected layer with 512 units and ReLU activation
  - Dropout with a rate of 0.5
  - Fully connected layer with 256 units and ReLU activation
  - Dropout with a rate of 0.5
  - Output layer with 69 units and softmax activation
- **Optimizer:** Adam with learning rate of 0.0001
- **Loss function:** Categorical Crossentropy
- **Training epochs:** 120 epochs

All models were trained using a training generator, with the number of steps per epoch determined by the formula train_generator.samples // batch_size. The validation data was also provided by a generator, with the number of validation steps determined by the formula valid_generator.samples // batch_size.

The workers parameter was set to 4 for all model training, and verbose mode was set to 1, providing a detailed log of the training process.

Link to each of the models:

<https://github.com/GaTechBrownLab/Rattray-2023-PLOSCompBio/tree/main/Models>
