# Supplementary material for: Machine learning identification of Pseudomonas aeruginosa strains from colony image data
Source: PLoS Comput Biol. 2023 Dec 13;19(12):e1011699. doi: 10.1371/journal.pcbi.1011699 (PMC10752536; doi:10.1371/journal.pcbi.1011699)
Supplement: S4 Table — We contrast validation and test accuracy for our trained ResNet model (see also Table 1) with accuracy metrics for radial basis function (RBF) SVMs trained on colony metric data (Fig 3 and 4) and on features extracted from the trained ResNet model. See methods for details of SVM models and feature extraction. Table A. ANOVA of Table 2. Tables B-C. Post-hoc pairwise tests (Tukey HSD with alpha = 0.05). (DOCX) [file pcbi.1011699.s005.docx]

**S4 Table.**  **Performance comparison with shallow learning (SVM) models.** We contrast validation and test accuracy for our trained ResNet model (see also Table 1) with accuracy metrics for radial basis function (RBF) SVMs trained on colony metric data (Figs 3 and 4) and on features extracted from the trained ResNet model. See methods for details of SVM models and feature extraction.

**Table A. ANOVA of Table 2.**

| **ANOVA** | **sum_sq** | **df** | **F** | **P-value** |
| --- | --- | --- | --- | --- |
| Validation Accuracy | 5268.1030 | 9.0 | 324.059336 | 3.608738e-11 |
| Test Accuracy | 7869.885293 | 3.0 | 909.536437 | 7.922344e-14 |

**Tables B-C. Post-hoc pairwise tests (Tukey HSD with alpha = 0.05).**

| Table B. Post Hoc Analysis for Validation Accuracy: | | | | | | |
| --- | --- | --- | --- | --- | --- | --- |
| **Group 1** | **Group 2** | **Mean Difference** | **p-adj** | **Lower** | **Upper** | **Reject** |
| Colony metric data, RBF SVM | ResNet-50 | 45.73 | 0 | 40.9195 | 50.5405 | TRUE |
| Colony metric data, RBF SVM | ResNet 50 feature extraction and RBF SVM | 19.4 | 0 | 14.5895 | 24.2105 | TRUE |
| ResNet 50 feature extraction and RBF SVM | ResNet-50 | 26.33 | 0 | 21.5195 | 31.1405 | TRUE |
| Table C. Post Hoc Analysis for Test Accuracy: | | | | | | |
| **Group 1** | **Group 2** | **Mean Difference** | **p-adj** | **Lower** | **Upper** | **Reject** |
| Colony metric data, RBF SVM | ResNet-50 | 56.094 | 0 | 52.5844 | 59.6036 | TRUE |
| Colony metric data, RBF SVM | ResNet 50 feature extraction and RBF SVM | 29.078 | 0 | 25.5684 | 32.5876 | TRUE |
| ResNet 50 feature extraction and RBF SVM | ResNet-50 | 27.016 | 0 | 23.5064 | 30.52 | TRUE |
